# The diagnostic value of dual-phase cone-beam CT during hepatic arteriography in transarterial chemoembolization for hepatocellular carcinoma

**DOI:** 10.1097/MD.0000000000024902

**Published:** 2021-03-26

**Authors:** Hiroki Higashihara, Keigo Osuga, Hiromitsu Onishi, Atsushi Nakamoto, Takahiro Tsuboyama, Noriyuki Tomiyama

**Affiliations:** a Department of Diagnostic and Interventional Radiology, Osaka University Graduate School of Medicine, 2-2 Yamadaoka, Suita; b Department of Radiology, Osaka Medical College, 2-7 Daigakucho, Takatsuki, Osaka, Japan.

**Keywords:** cone beam CT, diagnostic accuracy, hepatocellular carcinoma, liver, transarterial chemoembolization

## Abstract

To evaluate the diagnostic value of dual-phase cone beam CT during hepatic arteriography (CBCTHA) for hepatocellular carcinoma (HCC).

Thirty seven patients with unresectable HCC underwent the dual-phase CBCTHA prior to transarterial chemoembolization (TACE). Three blinded observers independently reviewed and compared the first phase CBCTHA images alone and the dual phase CBCTHA images. Diagnostic accuracy was evaluated by the alternative free-response receiver operating characteristic method (Area under the curve: Az value). Sensitivities were analyzed with the paired t test. The analysis was performed for overall HCCs, HCCs up to 1 cm and those larger than 1 cm.

For all HCCs and HCCs up to 1 cm, Az value and sensitivity showed no significant difference between the first-phase CBCTHA alone and the dual-phase CBCTHA (Az: 0.81 vs 0.88, *P* = .07, 0.79 and 0.85, *P* = .14, sensitivity: 0.61 and 0.73, *P* = .11, 0.41 and 0.52, *P* = .33, respectively). For HCCs larger than 1 cm, the mean Az value and sensitivity for the dual-phase CBCTHA were significantly higher than those for the first phase CBCTHA alone (Az: 0.96 vs 0.92, *P* = .008, sensitivity: 0.85 vs 0.75, *P* = .013, respectively).

The diagnostic accuracy of the dual-phase CBCTHA was superior to that of the first phase CBCTHA alone in the diagnosis of HCC larger than 1 cm.

## 1 Introduction

Transcatheter arterial chemoembolization (TACE) is the mainstay for palliative treatment of hepatocellular carcinoma (HCC) ineligible for surgery and ablation because of the tumor extent, multiplicity, or underlying cirrhosis.^[1–4]^

It is important to identify the precise size and number of HCCs during TACE. The usefulness of angiographic CT images obtained during the TACE procedure for the diagnosis of HCC.^[5–7]^ In particular, a combination of CT during arterioportography and dual-phase CT during hepatic arteriography (CTHA) contributes to the detection of HCC in during TACE.^[7]^

Cone beam CT (CBCT) technology using a flat-panel detector has become an alternative modality with CT-like images for a conventional CT scanner in angiography.^[8]^ CBCT has been reported as a useful modality not only to detect HCC but also to identify the feeding arteries during TACE.^[9–13]^ However, the precise diagnosis of HCC by CBCT is also necessary to use this software efficiently. The acquisition time of CBCT has been shortened to 5 to 10 seconds, and the acquired data can be transferred rapidly in 5 to 10 seconds. With these advancement, “dual-phase” CBCT during hepatic arteriography (CBCTHA) can be performed with only 1 contrast material injection, under the same scan timing as a multidetector CT-equipped angiography unit.

Corona enhancement often seen in the second phase of CTHA is one of the characteristic and most reliable findings to distinguish between hypervascular HCCs and arterioportal (AP) shunts.^[14]^ There has been a report about the incidence of the findings of corona enhancement of HCC in the dual-phase CBCTHA.^[15]^

We have reported about the diagnostic accuracy of CBCT with a combination of CT during arterioportography and the dual-phase CTHA for HCC compared with intravenous contrast-enhanced biphasic dynamic Multi-detector row CT (MDCT).^[16]^ In our previous study, the diagnostic accuracy of CBCT was equivalent to that of biphasic CT in the diagnosis of HCC. However, there have been no reports about the diagnostic accuracy and usefulness of adding the second phase CBCTHA to the first phase alone CBCTHA.

The purpose of this study was to evaluate the diagnostic value of the dual-phase CBCTHA for HCC compared with the first phase alone CBCTHA.

## 2 Materials and methods

### 2.1 Patient population

This study was approved as a retrospective one by the institutional review board of our institution. Our institutional review board waved the requirement for written, informed consent for participation to the present study. In our institution, the treatment strategy for all patients with HCC was decided by interventional radiologists, surgeons, and hepatologists in a consensus conference. From July 2009 to April 2010, TACE was performed for 244 patients, of whom 37 (26 men, 11 women; mean age, 71 years) were included in this retrospective study. Written informed consent about TACE for HCC was obtained from all patients. We selected the patients consecutively in the present study according to the following inclusion criteria:

1. TACE was judged as the appropriate treatment in the consensus conference;
2. no portal tumor invasion was shown on contrast enhanced CT; and
3. no aberrant hepatic artery was shown on contrast enhanced CT.

The patients who had the extrahepatic arterial tumor supply on CT were excluded in the present study.

### 2.2 Standard of reference for HCC

The 37 patients had a total of 100 hypervascular HCCs (1–9 tumors per patient). The maximal dimension of the HCCs ranged from 3 to 40 mm (mean, 13 **±** 7.3 mm) (Tables 1 and 2).

**Table 1:** Size of 100 HCC nodules.

Diameter (mm)	No. of HCC
≤5	13
6–10	27
11–20	49
≥21	11
Total	100

mean: 13 mm, SD: 7.3 mm HCC = hepatocellular carcinoma

**Table 2:** Distribution of patients based on number of HCC nodules.

No of HCC	Patients
1	10
2	13
3	7
4	2
5	2
7	1
9	2

HCC = hepatocellular carcinoma.

There was no pathological proof for HCC in all 37 patients. However, we decided the reference standard for HCC as descibed previously^[16]^ as follows:

1. the accumulation of iodized oil in the tumor on Lipiodol CT one week after TACE,
2. the tumor growth observed in non-treated segment at follow-up CT within 6 months after TACE.

The presence of HCCs was decided in consensus by 2 radiologists who had 22 and 12 years of experience in hepatobiliary imaging and were not included in the 3 readers for the image analysis (see below). The measurement of tumor size was also decided in consensus at the same time on the major axis of the axial image. In this study, the size of all HCCs was measured on only axial image. This is because there was no image in the another sectional image such as coronal and sagittal sections. In addition, thin-slice data such as 1 mm thickness was not left, and it was impossible to reconstruct a new multi-directional image for the measurement.

### 2.3 MDCT examination

All 37 patients were examined with dynamic contrast-enhanced CT with a 64-channel MDCT scanner (Aquilion 64, Toshiba, Tochigi, Japan) before TACE. Images were reconstructed in a section thickness of 5 mm with 5-mm intervals.

Non-ionic contrast material, a bolus of 100 ml iopamidol (370 mg I/ml, Iopamiron 370; Bayer Healthcare, Osaka, Japan) was administered intravenously via typically an antecubital vein at a rate of 3 to 4 ml/second with a power injector (Auto Enhance A-60; Nemotokyorindo, Tokyo, Japan).

For setting the adequate starting time of hepatic arterial phase scanning, an automatic bolus-tracking program (Real prep, Toshiba) was used. A circular region of interest (ROI) with an area of 50 pixels was placed in the aorta at the level of the celiac axis. The hepatic arterial phase scan started automatically 22 second after the threshold enhancement of 50 HU was reached in the aorta with the bolus-tracking program. The portal venous phase scan started 75 second just after contrast material injection.

### 2.4 TACE technique and CBCT examination

All TACE procedures underwent by the same flat-panel detector angiographic system (Allura Xper 127 FD20, Philips Healthcare). Firstly, a 4-Fr catheter was inserted via the femoral artery under the local anesthesia and digital subtraction angiography (DSA) was performed at the origin of celiac artery by a total of 20 ml iomeprol (Iomeron, Ezai, Tokyo, Japan) injected at a rate of 4 ml/second. Second, a coaxial 2-Fr microcatheter (Masters Parkwaysoft, Asahi Intecc, Seto, Japan) was placed and selective DSA (10 ml of iomeprol; flow rate, 2 ml/second) in the common hepatic artery was performed.

Dual-phase CBCTHA was subsequently performed for all patients. An acquisition techniques CBCTHA were as follows: total projection image, 312; total scanning angle, 207°; acquisition time, 10.4 seconds; matrix size, 256 × 256; effective field of view, 25 × 25 cm; voltage, 120 kv; current, 50 to 325 ma.

A total of 60 ml with 150 mgI/ml iomeprol was injected at a rate of 2 ml/second with a power injector through the microcatheter at the common hepatic or the proper hepatic artery. The scan for the first phase of CBCTHA started 15 seconds after, and the second phase of CBCTHA started 40 seconds after the injection of contrast material. CT-like images appeared automatically 10 seconds after scanning at a cross-sectional image to an equipped workstation (Xtra vision Interventional Workstation; Philips Healthcare). The axial images were reconstructed with a thickness of 5 mm with 5-mm intervals and used for image assessment.

Three interventional radiologists, who performed all TACE as a supervisor, assessed the conventional DSA and CBCTHA images and determined the number and location of HCCs and the feeding arteries that seemed to supply HCC.

From the microcatheter placed in the feeding arteries at the subsegmental or segmental level, a mixture of 1 to 4 ml iodized oil (Lipiodol; Terumo, Tokyo, Japan) and 10 to 40 mg epirubicin hydrochloride (Epirubicin: Nippon Kayaku, Tokyo, Japan) was administered according to the target tumor size and the hepatic area to be embolized. Additionally, porous absorbable gelatin particle (Gelpart: Nippon Kayaku, Tokyo, Japan) was injected gently until the blood flow stasis of the feeding arteries was obtained.

### 2.5 Image assessment

The image assessment was performed in the same way as previously reported.^[16]^ Images of the first phase alone CBCTHA and the dual-phase CBCTHA were interpreted independently in 2 sessions by 3 readers who had 10 to 14 years of experience in hepatobiliary imaging and did not participate in TACE. They were informed that all patients underwent TACE for HCC, but were blinded to the number and location of HCC. All images were interpreted on PACS viewers (Centricity Radiology RA 1000; GE Medical Systems, Milwaukee, WI, USA).

At first, each reader interpreted the first phase alone CBCTHA images and rated the location and size of the detected hepatic lesion on a schematic drawings of transverse sections using the following confidence scales: 0, no HCC present; 1, unlikely HCC; 2, equivocal HCC; 3, probably HCC; and 4, definite HCC.

Secondly, each reader assessed the dual-phase CBCTHA images (first phase and second phase) and rated in a similar fashion 3 weeks after the first session. To avoid learning bias, each reader viewed the dual-phase CBCTHA images in a different order.

**Figure 1:**
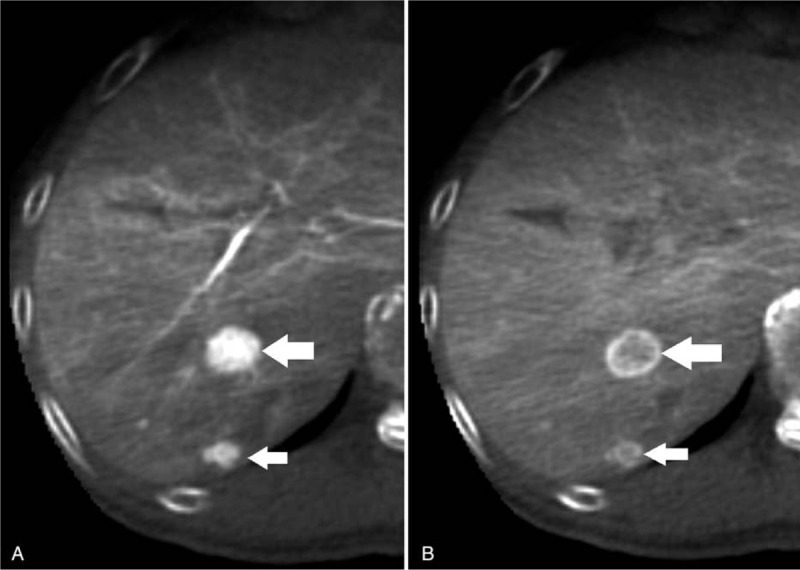
An 81-year -old man with hypervascular HCCs showing the typical CBCTHA finding. The first phase CBCTHA (A) demonstrated 2 hypervascular lesions with diameters of 1.6 cm and 0.8 cm (white arrow) in the subcapsular segment 8. The second phase CBCTHA (B) demonstrated washout of contrast material and rim enhancement, so called “corona enhancement” (white arrow). All readers rated them as HCCs with a high confidence level in both the first phase alone CBCTHA and the dual-phase CBCTHA.

We applied the typical findings of hypervascular HCC on CTHA images to that on the dual-phase CBCTHA images. The typical findings of hypervascular HCC was as follows;

1. the hyperattenuated lesion with round and well-defined shape,
2. the hyperattenuated lesion with nodular like enhancment,
3. the rim enhancement on the second phase CBCTHA (used for dual-phase only) (Fig. 1).^[17]^

### 2.6 Statistical analysis

The statistical analysis was also performed as previously described.^[16]^ We conducted alternative free-response receiver operating characteristic (AFROC) analysis on a tumor-by-tumor basis. An AFROC curve was prepared using a maximum-likelihood estimation program (ROCKIT 0.9B; C.E. Metz, University of Chicago, Chicago, IL, 1998) for fitting to each reader's confidence rating.^[18]^ We calculated the area under the AFROC curve (Az) for the estimation of diagnostic performance of methods and readers. Concerning the sensitivities and positive predictive values of each reader and each method with respect to the inclusion of HCCs allocated a confidence rating of 3 or 4 was regarded as positive. In addition to analysis of all HCCs, HCCs up to 1 cm in diameter and those larger than 1 cm in diameter were analyzed as subgroups.

The evaluation of statistical differences between the first phase alone CBCTHA imaging and the dual-phase CBCTHA imaging in Az, sensitivity, and positive predictive values was compared using the paired *t*-test. *P* < .05 was considered statistically significant.

Inter-reader variability of image interpretation among the 3 blinded readers was evaluated using the kappa value, so called the unweighted κ statistic. The binary value of 0 (not HCC) was assigned to HCCs with a confidence rating of 2 or less and the binary value of 1 (HCC) was assigned to HCCs with a confidence rating of 3 or greater. The κ values of 0.2 or less was regarded as slight agreements, 0.21 to 0.40 as fair, 0.41 to 0.60 as moderate, 0.61 to 0.80 as substantial, and 0.81 or more as almost perfect.^[19]^

## 3 Results

### 3.1 AFROC analysis

Table 3 showed the result of AFROC analysis. There was no significant difference in the mean Az value for the first phase alone CBCTHA and the dual-phase CBCTHA (Az _first__phase__alone_ = 0.81, Az _dual-phase_ = 0.88, *P* = .07) with regard to all HCCs. The mean Az value for the dual-phase CBCTHA was significantly higher than that for the first phase alone CBCTHA (Az _first__phase__alone_ = 0.92, Az _dual-phase_ = 0.96, *P* = .008) in the assessment of HCCs larger than 1 cm. The mean Az value for the dual-phase CBCTHA was not significant difference from that for the first phase alone CBCTHA (Az _first__phase__alone_ = 0.79, Az _dual-phase_ = 0.85, *P* = .14) in the assessment of HCCs up to 1 cm in diameter.

**Table 3:** Az values in detection of HCC.

	Reader				
HCC group	1	2	3	Mean	*P* value
All HCC					
early phase alone	0.83	0.79	0.81	0.81	.07
dual phase	0.90	0.89	0.85	0.88	
HCCs ≤1 cm					
early phase alone	0.78	0.77	0.82	0.79	.14
dual phase	0.83	0.87	0.84	0.85	
HCCs >1 cm					
early phase alone	0.95	0.92	0.90	0.92	.008
dual phase	0.98	0.96	0.94	0.96	

Az Values are area under the alternative free-response receiver operating characteristic curve HCC = hepatocellular carcinoma

### 3.2 Sensitivity and positive predictive value

Table 4 showed the result of sensitivity. The mean sensitivity of the dual-phase CBCTHA was significantly higher than that of first phase alone CBCTHA (*P* = .013) in HCCs larger 1 cm in diameter. There was no significant difference between these 2 groups in all HCCs and HCCs up to 1 cm in diameter (*P* = .11, *P* = .33, respectively).

**Table 4:** Sensitivity in detection of HCC.

	Reader				
HCC group	1	2	3	Mean	*P* value
All HCC					
Early phase alone	0.56 (56/100)	0.67 (67/100)	0.61 (61/100)	0.61	.11
Dual phase	0.75 (75/100)	0.72 (72/100)	0.71 (71/100)	0.73	
HCC ≤1 cm					
Early phase alone	0.27 (11/40)	0.48 (19/40)	0.48 (19/40)	0.41	.33
Dual phase	0.55 (22/40)	0.48 (19/40)	0.53 (21/40)	0.52	
HCCs >1 cm					
Early phase alone	0.75 (45/60)	0.80 (48/60)	0.70 (42/60)	0.75	.013
Dual phase	0.88 (53/60)	0.88 (53/60)	0.80 (50/60)	0.75	

Numbers in parentheses are actual numbers of lesions. HCC = hepatocellular carcinoma.

With regard to the difference of mean positive predictive value for the first phase alone CBCTHA and the dual-phase CBCTHA, there was no significant difference in all HCCs and the 2 subgroups (*P* = .06, *P* = .06, *P* = .08, respectively) (Table 5).

**Table 5:** Positive predictive values in detection of HCC.

	Reader				
HCC group	1	2	3	Mean	*P* value
All HCC					
Early phase alone	0.93 (56/60)	0.89 (67/75)	0.92 (61/65)	0.91	.06
Dual phase	0.97 (75/77)	0.96 (72/75)	0.95 (71/75)	0.96	
HCC ≤1 cm					
Early phase alone	0.85 (11/13)	0.76 (19/25)	0.85 (19/22)	0.82	.06
Dual phase	0.96 (22/23)	0.90 (19/21)	0.91 (21/23)	0.92	
HCCs >1 cm					
Early phase alone	0.94 (45/48)	0.92 (48/52)	0.93 (42/45)	0.93	.08
Dual phase	0.98 (53/54)	1.00 (53/53)	0.96 (48/50)	0.98	

Numbers in parentheses are actual numbers of lesions. HCC = hepatocellular carcinoma.

### 3.3 False-positive findings and false-negative findings

False-positive findings for HCCs were rated in 5 patients on the first phase alone CBCTHA and 4 patients on the dual-phase CBCTHA.

**Figure 2:**
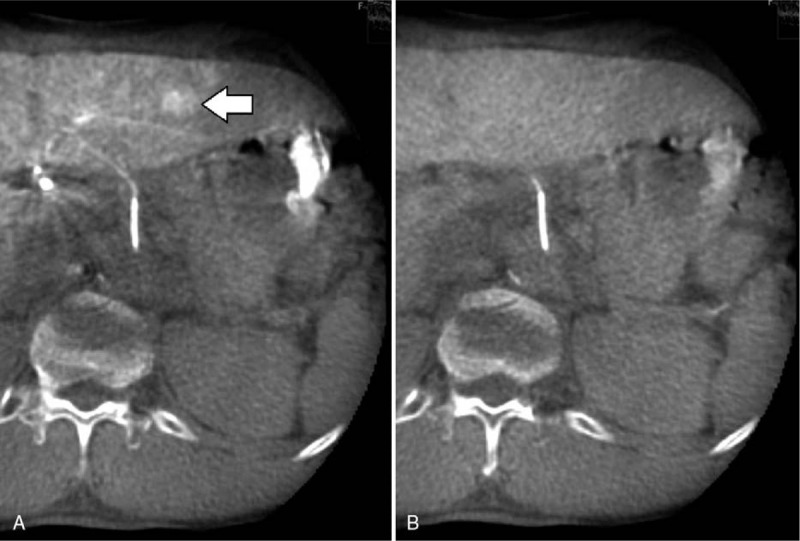
A 69-year-old man with a hypervascular HCC of the segments 3. The first phase CBCTHA (A) shows a slightly well-delineated hypervascular nodule with a diameter of 1 cm. On the second phase CBCTHA B), washout of contrast material, or corona enhancement was obscure. This nodule was not detected by any reader with a high confidence level.

**Figure 3:**
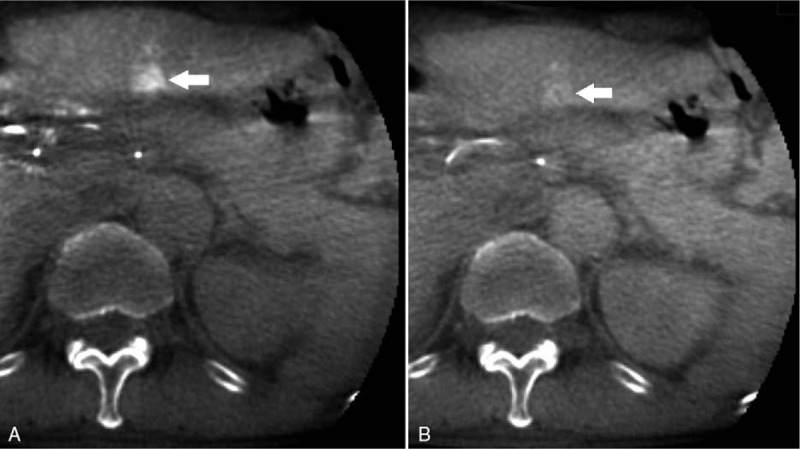
A 70-year-old man with a hypervascular HCC of the segment 3. On the first phase of CBCTHA (A), the nodule was demonstrated as a hypervascular lesion with a diameter of 1.2 cm. The nodule was well defined, but has an irregular shape. Therefore, all readers rated the score of the nodule as 2 with a low confidence level. On the second phase of CBCTHA (B), corona enhancement was well demonstrated. The rating score for dual-phase CBCTHA imaging was up to 3 in all readers with a high confidence level.

Eleven HCCs of 8 patients were not detected by any reader with a high confidence level on the first phase alone CBCTHA or the dual-phase CBCTHA (Fig. 2). On the first phase alone CBCTHA imaging, 20 HCCs of 16 patients were not detected by any reader with a high confidence level, while 9 of these HCCs could be detected on the dual-phase CBCTHA by at least 1 reader. All 3 readers could not detect 3 HCCs in 3 patients with a high confidence level on the first phase alone CBCTHA, while all these HCCs could be detected on the dual-phase CBCTHA by all readers (Fig. 3). Only 1 HCC was detected on the first phase alone CBCTHA but not detected on the dual-phase CBCTHA by all readers.

### 3.4 Inter-reader variability

The κ values of 3 readers were 0.57 for the first phase alone CBCTHA and 0.56 for the dual-phase CBCTHA. Moderate agreement was noted in the 3 readers with regard to the assessment of both the first phase alone CBCTHA and the dual phase CBCTHHA.

## 4 Discussion

“Corona enhancement,” which indicates the washout of contrast material around hypervascular HCCs on the late phase of single-level dynamic CTHA images, was first reported by Ueda et al.^[20]^ In hypervascular HCCs, perinodular hepatic sinusoids have collapse and continuity of intranodular and extranodular sinusoids interrupted by the capsule. Tumor blood drains into the surrounding liver parenchyma through the preserved portal vein within the capsule, and corona enhancement is demonstrated. Corona enhancement in CTHA is one of the characteristic findings of hypervascular HCCs, even though it is not seen in other liver tumors such as metastatic liver tumors.^[21,22]^ Since hypervascular pseudolesions such as arterioportal shunts (AP shunts) do not show corona enhancement on CTHA, corona enhancement is one of the most reliable diagnostic findings to discriminate between HCCs and AP shunts.^[14]^

In the present study, there was no significant difference between the first phase alone CBCTHA and the dual-phase CBCTHA in Az value, sensitivity, and positive predictive value among all HCCs. However, in HCCs larger than 1 cm, the mean Az value and sensitivity of the dual-phase CBCTHA were significantly higher than those of the first phase alone CBCTHA (Az 0.96 and 0.92, *P* = .008; sensitivity 0.85 and 0.75, *P* = .013, respectively). These results suggest that the dual-phase CBCTHA contributes to the detection and diagnosis of HCCs larger than 1 cm in diameter. Furthermore, 60% (12/20) of the false-negative HCCs on the first phase alone CBCTHA could be detected in the dual-phase CBCTHA. This improvement in diagnosis of HCC may be attributed to the finding of corona enhancement seen on the dual-phase CBCTHA.

Regarding the reason why the cut-off value was set to 1 cm for subgroup analysis, the slice thickness of dual phase CBCTHA used for tumor evaluation in this study was 5 mm in slice thickness with 5 mm interval, therefore there was a possible to be influenced by partial volume effect to diagnose HCC and accurate evaluation may not be possible for HCCs with up to 1 cm in diameter. Therefore, the subgroup analysis for 1 cm as a cut-off value also was considered to be adequate for the evaluation of diagnostic accuracy for HCC by dual-phase CBCTHA.

Miyayama et al reported that the detectability of corona enhancement in HCCs by the dual-phase CBCTHA was 88.7% (mean size: 17 ± 9 mm).^[15]^ In the present study, the mean sensitivity of the dual-phase CBCTHA in all HCCs and HCCs larger than 1 cm was 73% and 85%, respectively. If the sensitivity of HCCs in the dual-phase CBCTHA was regarded as the detectability of corona enhancement in HCCs, the result for the detectability of corona enhancement in Miyayama et al was higher than that for the sensitivity of HCCs in the dual-phase CBCTHA in the present study.^[15]^ One of the reasons for this discrepancy may be that they evaluated only the frequency of appearance of corona enhancement retrospectively. In the present study, the diagnostic capability for HCC in the dual-phase CBCTHA was evaluated by blind reading. Another reason may be that the mean size of HCCs in the present study (13 ± 7.3 mm) was smaller than that in the study by Miyayama et al (17 ± 9 mm).

The κ value for the dual-phase CBCTHA was 0.56 as moderate agreement. This result for the dual-phase CBCTHA suggests that any operator in TACE could correctly detect hypervascular HCC using the dual-phase CBCTHA.

This study has several limitations. First, this was a retrospective study with a small number of patients. Second, this study lacked the gold standard for the diagnosis of HCC such as pathologic proof. However, the combination of radiologic criteria for the findings of Lipiodol accumulation of HCC on CT 1 week after TACE and the findings of tumor growth on follow-up CT imaging within 6 months, should have minimized diagnostic errors in this study. Further investigation with a larger number of patients will be needed. Third, regarding the accuracy of tumor size measurement for the standard reference of HCC, the tumor size was measured on only axial image with MDCT. The evaluation for HCC might be more accurate based on the measurement of tumor size in multiple cross sections. However, there was no image in the another sectional image such as coronal and sagittal sections in this study. In addition, thin-slice data such as 1 mm thickness was not left, and it was impossible to reconstruct a new multi-directional image for the measurement.

However, in spite of these limitations, we could consider that this study has clarified the usefulness of the addition of second phase CBCTHA to the first phase CBCTHA.

In conclusion, the diagnostic accuracy for HCC larger than 1 cm in the dual-phase CBCTHA was higher than that in the first phase alone CBCTHA. The dual-phase CBCTHA is a useful additional diagnostic modality during TACE for HCC.

## Author contributions

**Conceptualization:** Hiroki Higashihara.

**Data curation:** Hiroki Higashihara, Hiromitsu Onishi.

**Formal analysis:** Hiroki Higashihara, Hiromitsu Onishi.

**Investigation:** Hiroki Higashihara, Hiromitsu Onishi, Atsushi Nakamoto, Takahiro Tsuboyama.

**Methodology:** Hiromitsu Onishi.

**Project administration:** Keigo Osuga.

**Supervision:** Noriyuki Tomiyama.

**Writing – original draft:** Hiroki Higashihara.

**Writing – review & editing:** Keigo Osuga.
